# Peripheral Blood-Based Gene Expression Studies in Schizophrenia: A Systematic Review

**DOI:** 10.3389/fgene.2021.736483

**Published:** 2021-10-13

**Authors:** Vipul Vilas Wagh, Parin Vyas, Suchita Agrawal, Tejaswini A. Pachpor, Vasudeo Paralikar, Satyajeet P. Khare

**Affiliations:** ^1^Symbiosis School of Biological Sciences, Symbiosis International (Deemed University), Pune, India; ^2^The Psychiatry Unit, KEM Hospital and KEM Hospital Research Centre, Pune, India; ^3^Department of Biodiversity, MES Abasaheb Garware College, Pune, India

**Keywords:** schizophrenia, genomics, gene expression, peripheral blood, biomarkers

## Abstract

Schizophrenia is a disorder that is characterized by delusions, hallucinations, disorganized speech or behavior, and socio-occupational impairment. The duration of observation and variability in symptoms can make the accurate diagnosis difficult. Identification of biomarkers for schizophrenia (SCZ) can help in early diagnosis, ascertaining the diagnosis, and development of effective treatment strategies. Here we review peripheral blood-based gene expression studies for identification of gene expression biomarkers for SCZ. A literature search was carried out in PubMed and Web of Science databases for blood-based gene expression studies in SCZ. A list of differentially expressed genes (DEGs) was compiled and analyzed for overlap with genetic markers, differences based on drug status of the participants, functional enrichment, and for effect of antipsychotics. This literature survey identified 61 gene expression studies. Seventeen out of these studies were based on expression microarrays. A comparative analysis of the DEGs (*n* = 227) from microarray studies revealed differences between drug-naive and drug-treated SCZ participants. We found that of the 227 DEGs, 11 genes (*ACOT7, AGO2, DISC1, LDB1, RUNX3, SIGIRR, SLC18A1, NRG1, CHRNB2, PRKAB2, and ZNF74*) also showed genetic and epigenetic changes associated with SCZ. Functional enrichment analysis of the DEGs revealed dysregulation of proline and 4-hydroxyproline metabolism. Also, arginine and proline metabolism was the most functionally enriched pathway for SCZ in our analysis. Follow-up studies identified effect of antipsychotic treatment on peripheral blood gene expression. Of the 27 genes compiled from the follow-up studies *AKT1, DISC1, HP*, and *EIF2D* had no effect on their expression status as a result of antipsychotic treatment. Despite the differences in the nature of the study, ethnicity of the population, and the gene expression analysis method used, we identified several coherent observations. An overlap, though limited, of genetic, epigenetic and gene expression changes supports interplay of genetic and environmental factors in SCZ. The studies validate the use of blood as a surrogate tissue for biomarker analysis. We conclude that well-designed cohort studies across diverse populations, use of high-throughput sequencing technology, and use of artificial intelligence (AI) based computational analysis will significantly improve our understanding and diagnostic capabilities for this complex disorder.

## Introduction

Schizophrenia is a multifactorial disorder with 1.13 million incident cases globally. Schizophrenia has claimed 12.66 million disability-adjusted life years up to 2017 (He et al., 2020). The death rate in schizophrenia patients is much higher when compared to healthy individuals (Saha et al., 2007; Lomholt et al., 2019). Schizophrenia claims millions of lives globally every year, with cardiovascular diseases as a leading cause of death followed by suicide, and respiratory and cancer-related disorders (Bushe et al., 2010). The current treatment for SCZ includes a combination of antipsychotics and behavioral therapy. The development of second generation antipsychotics has lead to effective treatment for both positive and negative symptoms of SCZ (Zhang et al., 2013; Mauri et al., 2014; Gründer et al., 2016; Chen and Nasrallah, 2019). Introduction of third generation antipsychotics has also contributed in lowering extrapyramidal side effects (Stepnicki et al., 2018). Even though the antipsychotics have evolved into relatively specific drugs for the clinical pathology, these drugs still happen to be part of symptom management therapy. The cure for SCZ still lies in the future; however, upon timely intervention of the available treatment, affected individuals can live a relatively symptom-free life.

Schizophrenia, as a disorder, has been known to humankind for over 100 years. The complexity of the disorder is known and has been highlighted since its identification. Eugene Bleuler proposed the term “Schizophrenia” in 1908, describing it as a group of disorders existing together and leading to the disoriented state of mind (Heckers, 2011; Moskowitz and Heim, 2011). These co-existing disorders are now categorized into positive and negative symptoms. Hallucinations, delusion and derailment of speech are positive symptoms while diminished emotional expression and symptoms similar to it are categorized as negative ones. The presence of positive and negative symptoms forms the basis of current diagnostic methods laid by International Classification of Diseases (ICD) (World Health Organization, 2004) and Diagnostic and Statistical Manual of Mental Disorders (DSM) (American Psychiatric Association, 2013). DSM being specific to psychiatric disorders is widely accepted for the diagnosis of the SCZ. The recent edition of DSM (DSM-5) has eliminated the subtypes of SCZ owing to their limited diagnostic ability and identifies SCZ as the single disorder (American Psychiatric Association, 2013).

Even with the criteria laid by DSM and ICD for the diagnosis of psychiatric disorders the misdiagnosis in psychiatry is quite common (Mukherjee et al., 1983; Shen et al., 2018; Tzur Bitan et al., 2018; Coulter et al., 2019). Also, according to these guidelines a person is required to have a set of behavioral symptoms for a specific period, which generally exceeds 1 month for both DSM as well as ICD. Further, the presence of symptoms needs to be profound enough to be even considered for diagnosis. The time required for the diagnosis may delay the necessary early intervention of the treatment. Early and reliable diagnosis may help to reduce the vulnerability, rate of conversion to psychosis, time to remission, incidence of the disorder, economic burden on the society and premature deaths (Bahn et al., 2011). Identification of biomarkers can aid current diagnostic process for accurate and timely diagnosis of the SCZ. However, extreme care should be taken during the development and use of the possible biomarkers for schizophrenia, as it may lead to stigmatization as well as other ethical dilemmas.

Brain imaging methods have shown anatomical changes in whole brain as well as specific regions in SCZ patients. Smith and co-workers carried out the first imaging study using Magnetic Resonance Imaging (MRI) in 1984 (Smith et al., 1984). Since then, a plethora of imaging studies have emerged and have identified structural changes in the brain associated with SCZ (Andreasen et al., 1990; Staal et al., 1998, 2000; Gur et al., 1999; Levitt et al., 1999; Downhill et al., 2000; Sanfilipo et al., 2000). The findings of imaging studies are limited to the identification of structural and volumetric changes in the brain (Shenton et al., 2001). The evolution of MRI into functional MRI (fMRI) has given a new hope for the discovery of imaging biomarkers for psychiatric disorders (Du et al., 2012; Yoon et al., 2012; Su et al., 2013; Koch et al., 2015). Identification of functional changes in correlation with the loss of cognitive functions may help to differentiate the SCZ phenotype from others. However, for the heterogeneous disorder like SCZ where affected individuals experience different sets of symptoms with different intensity, the structural and volumetric changes induced in the brain may not be disorder-specific (Linden, 2012). Lack of reproducibility, smaller sample size, low feasibility of clinical application and higher cost, limit the use of imaging analysis as a corroboratory diagnostic tool for behavioral disorders such as SCZ (Linden, 2012).

A combination of genetic and environmental factors are suspected to be responsible for the development of SCZ (Tsuang, 2000; Howes et al., 2017). Genome-Wide Association Studies (GWAS) have identified single nucleotide polymorphisms (SNPs) (Ripke et al., 2014) and copy number variations (CNVs) (Marshall et al., 2017) in a large number of quantitative trait loci (QTLs). However, these genetic variations altogether explain 70–80% of heritability^1^ (Hilker et al., 2018). The risk of developing SCZ in the offspring of affected and non-affected identical twin suggests a role of gene-environment interaction in the development of SCZ (Gottesman and Bertelsen, 1989; Kringlen and Cramer, 1989). Environmental factors often affect genes by covalently modifying (methylating) the DNA thus affecting the gene expression.

Recently various studies have attempted epigenome wide changes such as DNA methylation for their association with the disorder (Numata et al., 2014; Wockner et al., 2014; Jaffe et al., 2015; Viana et al., 2017). However, lack of accessibility of brain tissue for epigenetic studies has resulted in a limited number of studies for psychiatric disorders. Non-target tissues such as peripheral blood have also been used for epigenome profiling. These studies have identified differentially methylated loci in SCZ. Site specific DNA methylation changes using peripheral blood have also been explored as potential biomarkers (Ikegame et al., 2013; Cheng et al., 2014; Nabil Fikri et al., 2017; Nour El Huda et al., 2018).

In other complex diseases such as diabetes (Nathan et al., 2009) and cancer (Deras et al., 2008; Esserman et al., 2017), blood-based biomarkers are already in use in clinical settings. However, the use of peripheral blood as a tissue for biomarker discovery in behavioral disorders has been debated since long. But it is well evident from the recent literature that SCZ phenotype is associated with molecular changes in the non-target tissue such as blood (Levin et al., 2010; Montano et al., 2016; Gilabert-Juan et al., 2019). The presence of inflammatory markers in the brain suggests the existence of a blood-brain relationship (Black and Miller, 2015; Trovão et al., 2019). Also, the immune hypothesis (Kinney et al., 2010; Muller and Schwarz, 2010) suggests the onset of the SCZ may begin with the crossing of the blood-brain barrier by immune cells from the peripheral tissue (Capuron and Miller, 2011; Khandaker and Dantzer, 2016; Van Kesteren et al., 2017). A transcriptome-wide mega-analyses has revealed a correlation between blood and brain gene expression changes in SCZ (Hess et al., 2016). Discovery of SCZ specific molecular markers from peripheral blood may not necessarily indicate the cause and effect relationship. However, the specificity and sensitivity of the markers can be exploited for their potential use as biomarkers.

With the recognized scope for identification of molecular biomarkers, RNA and protein expression studies in peripheral blood for Schizophrenia are on the rise. The surge in the discovery for protein-based biomarkers comes from their important role as functional molecules in cellular processes. The deregulated proteome in SCZ may be a result of underlying pathophysiology associated with the disorder (Martins-de-Souza et al., 2009; Reis-de-Oliveira et al., 2020). A few proteomic studies have revealed the dysregulated pathways associated with immune system in the peripheral blood of SCZ (Li et al., 2012; Ezeoke et al., 2013; Goldsmith et al., 2016). Further, proteomic studies with varied approaches have also identified differentially expressed proteins with a significant diagnostic potential for SCZ (Schwarz et al., 2010; Nascimento and Martins-De-Souza, 2015; Comes et al., 2018). Even with the application of mass spectrometry, the identification and quantification of low abundance protein still remains a limitation (Pradet-Balade et al., 2001; Chandramouli and Qian, 2009). One might argue that already developed tools like ELISA can be used for protein biomarker discovery in SCZ. However, the ELISA based techniques rely on the abundance of target protein present in the sample and availability of specific antibodies for the detection (Del Campo et al., 2015). In contrast, the nucleic acids such as messenger ribonucleic acid (mRNA) are much more sensitive to the genomic and epigenomic changes. For the complex disorder like SCZ where genetic and epigenetic mechanisms are suspected to play an important role in the development and progression of the disorder, gene expression profiling may be a feasible option for the biomarker discovery.

Recent transcriptomic studies using peripheral blood have shown a significant correlation between gene expression profile and the clinical features of SCZ (Bousman et al., 2010b; Wu et al., 2016; Zheutlin et al., 2016). Focusing on these gene expression changes may shed light on molecular pathways involved in the development of the disorder (Middleton et al., 2005; Wu et al., 2016). Researchers have also identified similarities and dissimilarities in gene expression profiles in peripheral blood of Schizophrenia, Bipolar disorder (BPD) and Major depressive disorder (MDD) (Cattane et al., 2015; Miyamoto et al., 2020). Altogether, the findings suggest that the sensitive and specific nature of gene expression changes in the peripheral tissue such as blood can be exploited for the development of diagnostic, prognostic and predictive biomarkers.

Considering the prospects of application of differentially expressed genes into the clinics, here we chose to review the gene expression studies using peripheral blood in SCZ. We have used these studies to summarize the methodologies and their findings. Further, we also discuss the limitations of the current approaches and discuss possible solutions to overcome them.

## Methodology

### Design

We performed a systematic review of peripheral blood gene expression biomarkers for SCZ. The guidelines set by Preferred Reporting Items for Systematic Reviews and Meta-Analyses (PRISMA) were followed to conduct this review (Moher et al., 2009). Covidence software was used for screening of the research articles (Covidence systematic review software, Veritas Health Innovation).

### Search Strategy

**Figure 1:**
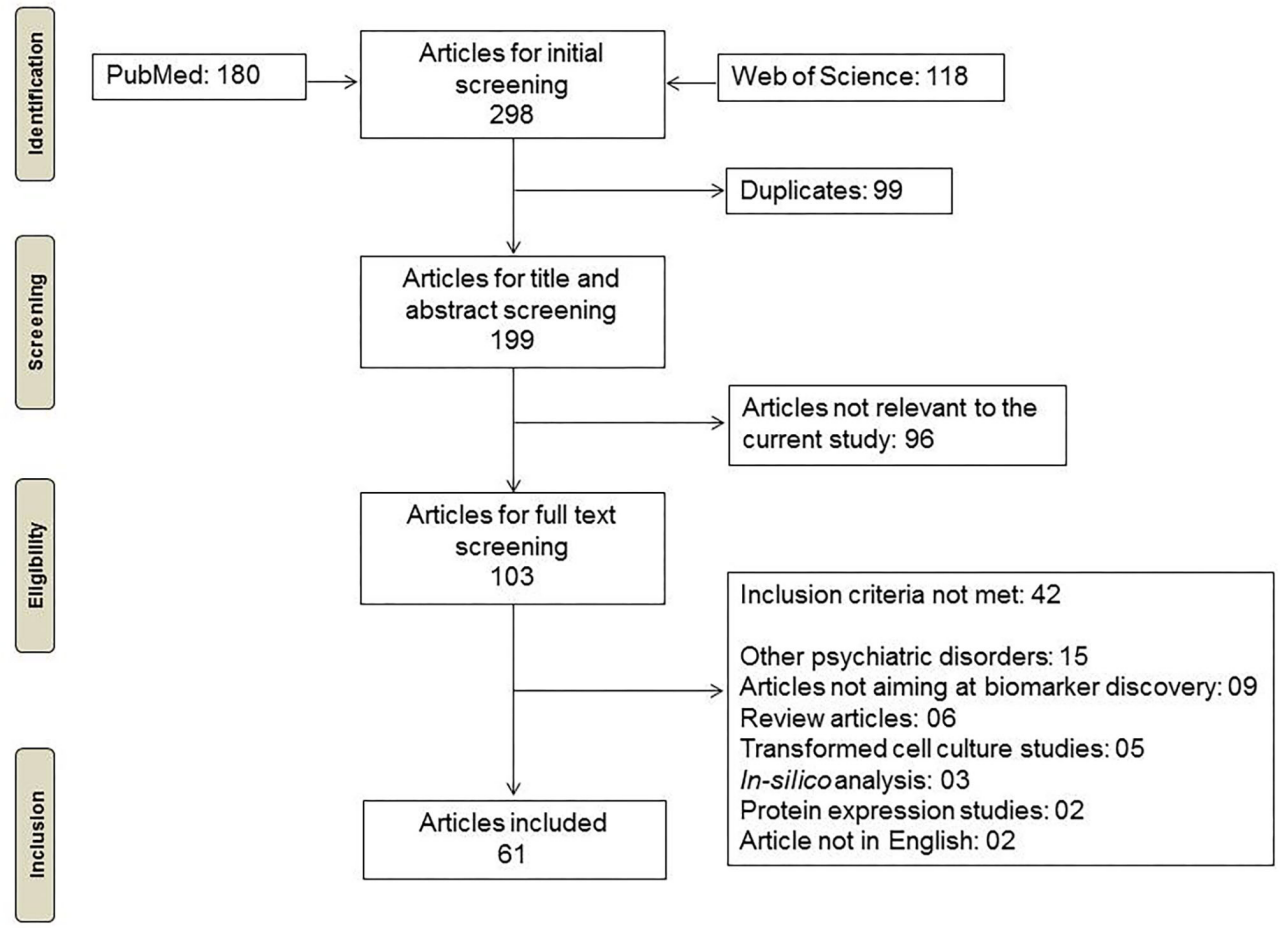
Flowchart depicting the methodology for inclusion of articles.

Research studies were identified from PubMed and Web of Science electronic databases using the keywords “Gene expression,” “Peripheral blood,” “Biomarkers” and “Schizophrenia” or “Schizophrenia spectrum.” We included research articles published until February 2021 for further screening (Figure 1).

### Eligibility

We included peer-reviewed original research articles aiming at identification of differentially expressed genes in peripheral blood of SCZ participants. Studies with diagnostic schemes other than DSM and ICD were also included. Cross-sectional and follow-up studies with drug-naive and drug treated participants were also included. We did come across studies aiming at identification of non-coding RNAs (miRNA, lncRNA, circRNA) from peripheral blood in association with SCZ. However, studies on lncRNA (Chen et al., 2016; Melbourne et al., 2018; Fallah et al., 2019; Safari et al., 2019) and circRNA (Yao et al., 2019) were excluded as their number was not significant for a systematic review. Further, miRNA dysregulation in SCZ and its application as biomarker has already been reviewed and hence these studies were excluded as well (Beveridge and Cairns, 2012; Wang et al., 2014; Liu et al., 2017).

Research studies on psychiatric disorders other than SCZ, gene expression studies using transformed peripheral blood mononuclear cells (PBMCs), protein expression studies and *in silico* analysis without any supportive wet lab validation of the markers, were excluded from the review. Research articles published in languages other than English were not considered for the review.

### Data Extraction

In addition to the information about authors and year of publication, participants' information such as demographic details, number of participants, medication status, detection methods, potential biomarkers, significant findings and type of study were also extracted for all the studies (Supplementary Table 1). We further narrowed down our focus on microarray based transcriptomic studies due to their exploratory approach. Custom microarray-based studies and those with no available information on medication status of the participants, were excluded from further analysis. Significantly differentially expressed genes (*p* < 0.05) from the microarray studies were compiled. The gene symbols were verified against the approved symbols by HUGO gene nomenclature committee (HGNC) using the Multi-Symbol checker tool (Braschi et al., 2019). The differentially expressed genes (DEGs) between cases and controls were categorized based on drug status (“drug-treated” or “drug-naïve”) of the participants.

### Data Analysis

We performed a functional enrichment analysis on DEGs using online tools such as g:Profiler (Raudvere et al., 2019) and Database for Annotation, Visualization, and Integrated Discovery (DAVID) (Dennis et al., 2003). To observe the similarities between the DEGs compiled in this literature survey and findings of genetic and epigenetic studies, we queried SZDB2.0 (Wu et al., 2020). A comparative analysis was performed between drug naïve and drug treated groups by plotting a Venn diagram of DEGs. Genes in each category were also subjected to gene ontology and pathway enrichment analysis as described earlier. We also identified studies which have evaluated biomarkers for their diagnostic potential. The potential biomarkers, methods used for evaluation and their results in terms of accuracy, sensitivity, specificity, etc. were compiled for these studies. Further, from the previously identified sixty one gene expression studies, we identified those aimed at evaluating the effect of antipsychotics on gene expression. The DEGs and the reported change in their expression levels before and after antipsychotic treatment were tabulated (**Table 5**).

## Results and Discussion

### Screening of Peripheral Blood-Based Gene Expression Studies

**Figure 2:**
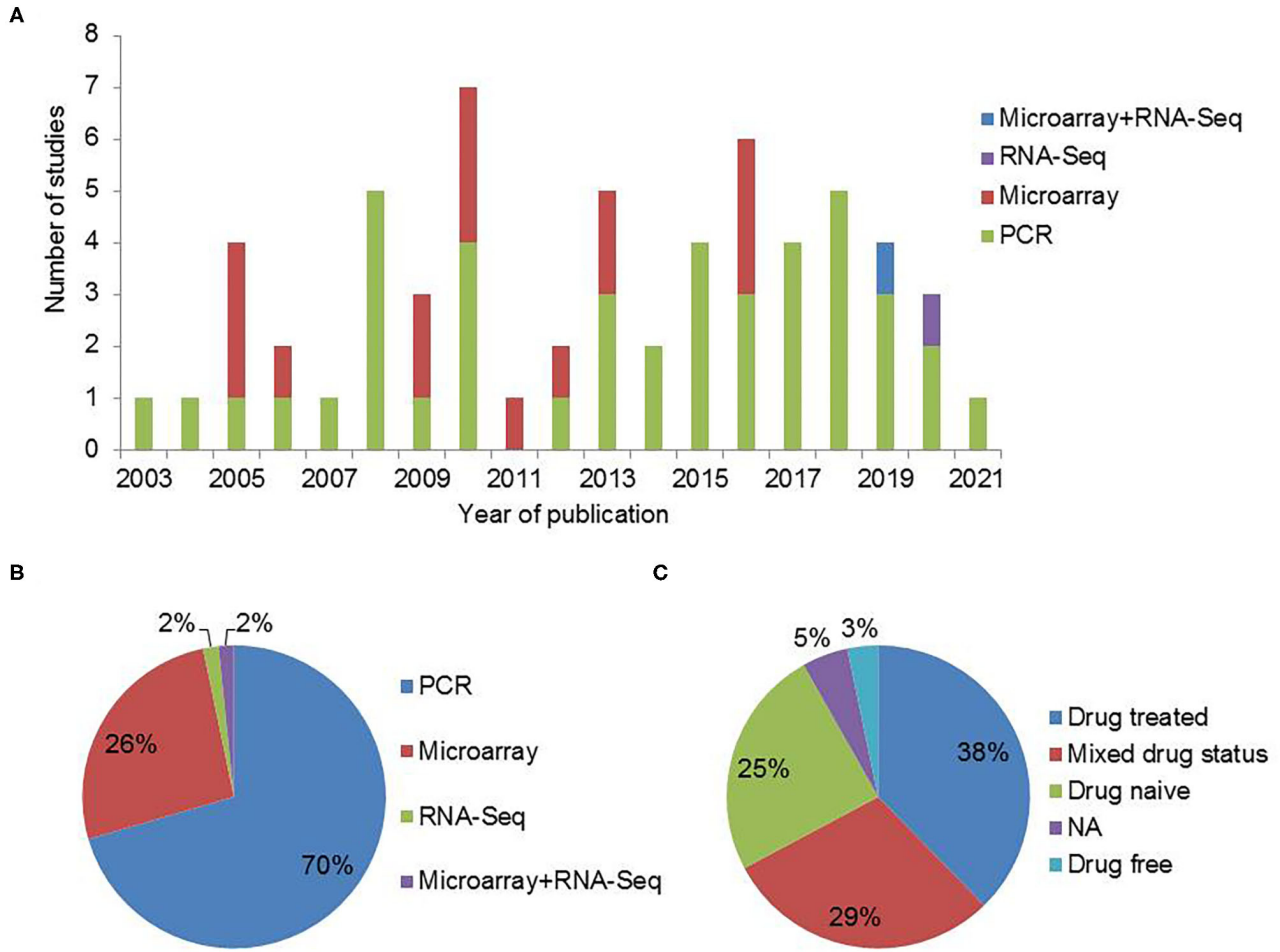
Peripheral blood based gene expression studies in schizophrenia. **(A)** Gene expression studies for SCZ published over the years. **(B)** An overview of the methods used for gene expression analysis. **(C)** Type of participants included in the gene expression studies for SCZ (NA: Information not available).

We identified 180 studies from PubMed and 118 studies from Web of Science. After removal of duplicates and filtering out the articles based on abstract screening, 103 articles were retained. Full text screening for inclusion criteria resulted in 61 research articles relevant to the topic of interest (Figure 1). Most of these 61 studies used DSM for diagnosis of SCZ. Thirty seven of the 61 studies provided explicit information about ethnicity. Also, 47 of the shortlisted studies were cross-sectional in nature whereas others were follow-up studies (Supplementary Table 1). Peripheral blood based biomarker studies for schizophrenia showed consistent increase over the past two decades (Figure 2A). Most of the studies take PCR based targeted approach. Among transcriptomic studies, the number of microarray based articles was significantly larger than the RNA-Sequencing based ones. Most of the PCR based studies validated previously reported DEGs in their respective cohorts (Figure 2B). The effect of antipsychotics on peripheral gene expression appears to be well recognized as a quarter of the studies screened included drug-naive participants to identify potential diagnostic and clinical state biomarkers (Figure 2C).

### Transcriptomic Studies for Schizophrenia

We came across 17 microarray studies published during 2005–2019. For an unbiased comparison of the findings, studies with custom microarray were excluded (Zvara et al., 2005; Bowden et al., 2006). The resulting 15 transcriptomic studies (Table 1) majorly differed from each other based on the medication status of the SCZ participants. Study with no available information on medication status of the participants, was excluded from the comparative analysis (Middleton et al., 2005). Thus the resulting 14 studies with the information available for medication status of the participants were used for the comparative analysis described further in this review.

**Table 1:** Summary of 15 microarray studies included for the preliminary analysis.

**Author**	**Ethnicity sample size**	**Demographic details**	**Psychiatry tools**	**Medication status**	**Findings**	**Study type**
Tsuang et al. (2005)	All Han Chinese except 3 Caucasian controls SCZ = 30, BPD = 16, CT = 28	Age	DSM-IV	Drug treated	• The putative biomarkers identified in the study were able to differentiate between SCZ, BPD and CT samples with an accuracy of 95%-97%.	Cross-sectional
Middleton et al. (2005)	Ethnicity=NA, SCZ = 33, CT = 33 (Discordant sibling pairs)	Age and Sex	DSM-IV	NA	• Genes involved in Immune/inflammatory response had significant higher expression.	Cross-sectional
					• Altered expression of SMDF suggests the role of NRG signaling in SCZ	
	BPD = 5, CT = 5 (Discordant sibling pairs)				• Genes involved in fatty acid and lipid metabolism were differentially expressed in both SCZ and BPD	
					• Cytogenomic analysis revealed distinct cytogenomic profiles for SCZ and BPD	
Glatt et al. (2009)	European, Hispanic, African and Asian SCZ = 13, BPD = 11, CT = 8	Age, sex, smoking, medication, number of psychotic episodes, ancestry	DIGS	Drug treated	• 18 genes in BPD and 8 genes in SCZ and 15 genes in both the groups were differentially spliced compared to controls.	Cross-sectional
					• Frequent exon inclusion and/or over expression was associated with psychosis.	
					• Exomic and spliceomic profile identified transcripts specific to psychiatric disorders	
Kuzman et al. (2009)	Caucasian SCZ = 32, CT = 32	Age, sex, duration of illness, educational years, family history	DSM-IV PANSS	Drug Naïve on recruitment	• 180 genes were found to be differentially expressed in association with SCZ	longitudinal study with up to 2 year follow-up
	Follow-up study: SCZ = 12 (achieved full remission)			Drug treated for follow-up study	• DAAM2 expression levels correlated with the progression of the disorder	
Takahashi et al. (2010)	Ethnicity=NA SCZ = 52, CT = 49	Age, sex, medication status and age at onset	DSM-IV SCID-I	Drug Naïve and Drug Free (Participants who stopped taking medication for more than 8 weeks)	• Microarray analysis revealed 11 genes from the differentially expressed in association with SCZ.	Cross-sectional
					• The ANN differentiated schizophrenia samples from the controls with the accuracy of 87.9%.	
Bousman et al. (2010b)	European, African, Hispanic and Asian SCZ = 13, BPD = 6, CT = 8	Age, sex, ancestry, education, smoking, substance abuse, medication, psychosis history	DSM-IV SAPS SANS	Drug treated	• A positive association was observed between *UBE2K, SIAH2* and SAPS score while a negative association was observed with USP2	Cross-sectional
Bousman et al. (2010a)	**San Diego Cohort:** European, African, Hispanic, Asian SCZ = 13, BPD = 9, CT = 8	Age, sex, ancestry, education, smoking, substance abuse, current medication and history of psychosis	San Diego Cohort: DIGS	Drug treated	• Ubiquitin proteasome pathway (UPS) was one of the top dysregulated pathways was associated with BPD and Psychosis in both the cohorts	Cross-sectional
	**Taiwan Cohort:** Han Chinese SCZ = 11, BPD = 14, CT = 16		Taiwan Cohort: DSM-IV		• Glutamate metabolism and Chondroitin sulfate biosynthesis pathways were one of the top dysregulated pathways in SCZ group for San Diego and Taiwan cohort respectively	
Glatt et al. (2011)	Caucasian and African-American SCZ = 7, Siblings = 7, CT = 12	Age, sex, ancestry, education, current medication, history of psychosis and IQ	DIGS	Drug treated	• The dysregulated genes identified in both SCZ affected individuals and their siblings were related to nucleosome and histone structure and function.	Cross-sectional
					• The differential expression of genes in healthy siblings may indicate the protective effect of these genes against SCZ.	
Maschietto et al. (2012)	Ethnicity = NA SCZ = 28, CT = 10	Age, sex, duration of illness and age of onset	Diagnostic scale = NA BPRS	Drug treated	• Linear Discriminant Analysis identified 6 genes which differentiated SCZ samples from controls with the sensitivity of 89.3% and specificity of 90%.	Cross-sectional
Gardiner et al. (2013)	Caucasian SCZ = 114, CT = 80	Age, sex, age of onset, family history, duration of illness, medication and duration of medication	ICD-10	Drug treated	• Microarray analysis revealed altered expression of 164 genes (59 up-regulated and 105 down-regulated) in SCZ affected individuals compared to healthy controls.	Cross-sectional
					• In silico analysis identified the involvement of immune function pathways in association with SCZ.	
					• PCR validation confirmed the differential expression of the*AGO2, MEF2D, EVL, PI3, S100A12* and *DEFA4* in association with SCZ.	
Kumarasinghe et al. (2013)	Sinhalese SCZ = 10, CT = 11	Age, sex, family history, duration of illness, medication and duration of medication	DSM-IV BPRS	Drug Naïve	• Microarray analysis revealed altered expression of (208 up-regulated, 416 down-regulated) prior to antipsychotic treatment.	Follow-up
					• Post antipsychotic treatment, only 106 genes were altered and 67 genes had no change in the expression levels.	
Xu et al. (2016)	Han Chinese	Age, sex, age of onset and duration of illness	DSM-IV, PANSS	Drug Naive and drug treated	• Microarray analysis identified 84 differentially expressed genes (82 up regulated, 2 down regulated)	Follow-up
	Microarray analysis: SCZ = 18, CT = 12					
					• Olfactory transduction pathway was found to be the most enriched pathway for the genes obtained from co-expression analysis	
	qRT-PCR validation: SCZ = 38, CT = 48					
					• A variation in the gene expression levels was observed on antipsychotic treatment	
Zheutlin et al. (2016)	**Swedish cohort:** SCZ = 36, SCZ Twins = 34, CT = 81 BPD = 23, BPD Twins = 16	Age, sex, zygosity	DSM-IV SCID-IV, CVLT, SANS and SAPS	Drug treated	• Microarray analysis identified 41 genes differentially expressed in SCZ compared to controls and siblings.	Cross-sectional
	**Finnish cohort:** SCZ = 18, SCZ Twins = 18, CT = 37				• Most of the DEGs identified contained common or de novo mutations associated with SCZ	
(Wu et al., 2016)	Ethnicity = NA SCZ = 47, CT = 49	Age, sex, age of onset, types of antipsychotics	ICD-10, RBANS COWAT, WASI, LNS, WTAR	Drug treated	• The transcriptome profiling in SZ and cognitive subtypes were characterized by the up- regulated pathways involved in immune, energy metabolism and down-regulation of the pathways involved in neuronal signaling (e.g., WNT in SZ/CD and ERBB in SZ)	Cross-sectional
Gilabert-Juan et al. (2019)	Spanish descent Cohort 1-SCZ (chronic) = 30, CT = 15 Cohort 2-SCZ (first-episode) = 124, CT = 54	Age, sex, ethnicity, medication, clinical condition, patient condition and family history	Cohort 1: DSM-IV, SCID-1, PANSS	Cohort—drug treated	• *EIF2D* and *TOX* were differentially expressed in SCZ affected individuals from cohort 1	Follow-up
					• *EIF2D* expression levels were also altered in drug naive SCZ individuals	
					• *EIF2D* and *TOX* could differentiate the SCZ with the accuracy of 86.5% from control samples	
			Cohort 1: DSM-IV, PANSS	Cohort 2—drug Naive	Expression levels of • *EIF2D* significantly varied in cohort 1, with progression of the disorder	

*The details of the studies including significant findings are mentioned in the table*.

*CT, Healthy Controls; SCZ, Schizophrenia affected participants; NA, not available. Psychiatry tools such as DSM-IV, Structured Clinical Interview for Axis I Disorders (SCID-I), Structured Clinical Interview for DSM-IV (SCID-IV), ICD-10 and Diagnostic Interview for Genetic Studies (DIGS) are used for diagnosis of the psychiatric disorders. While Scale for Assessment of Negative Symptoms (SANS), Scale for Assessment of Positive Symptoms (SAPS), Positive and Negative Syndrome Scale (PANSS), Brief Psychiatric Rating Scale (BPRS) are use to record the severity of the symptoms. The Wechsler Abbreviated Scale of Intelligence (WASI) and Wechsler Test of Adult Reading (WTAR) are intelligence quotient predicting scales. While, Repeatable Battery for Assessment of Neuropsychological Status (RBANS), Controlled Oral Word Association Test (COWAT), California Verbal Learning Test (CVLT) and Letter-Number Sequencing test (LNS) are used for neurocognitive assessment*.

### Genetic and Epigenetic Changes, and DEGs

A total of 227 DEGs were reported with respect to healthy controls in the 14 shortlisted microarray studies. Genetic studies have identified several SNPs and CNVs associated with the SCZ phenotype. These polymorphisms and variations may result in the differential expression of genes across all the tissues. To observe the similarities between the DEGs identified from this literature survey and previously reported GWAS findings, we queried SZDB2.0 (Wu et al., 2020). Interestingly, *CTNNA1, SATB2, SPATA31D1*, and *SLC45A1* from this literature survey were also reported by Psychiatric Genomics Consortium (PGC2) (Ripke et al., 2014) and Clozapine clinic UK (CLOZUK) GWAS (Pardiñas et al., 2018). Similar to the GWAS, Marshall et al., identified 16 independent CNV loci associated with SCZ (Marshall et al., 2017). Out of the genes that were affected by CNVs, *PRKAB2*, and *ZNF74* were common to 227 genes. Marshall et al. have also reported an elevated risk for SCZ with the gain of CNVs in these genes (Marshall et al., 2017). Other than GWAS and CNV studies, genetic linkage and association studies have linked *SLC18A1, DISC1, NEUROG1, PRODH, CCKAR, DAO, NRG1, DAOA, AKT1, NTF3, CHRNB2, CHI3L1, CHGA, ACKR1*, and *DGCR6* common to the 227 DEGs to the SCZ phenotype (Lewis et al., 2003; Allen et al., 2008; Sun et al., 2008; Ng et al., 2009). Further, an overlap of 54 genes was observed between 227 DEGs and genes identified by exome analysis. Two of these 54 genes viz. *CTNNA1, SPATA31D1* were also identified by GWAS studies.

In addition to the genetic changes, we also explored DNA methylation changes reported in peripheral blood of SCZ participants. Similar to the genetic analysis, peripheral blood methylation studies in SZDB2.0 were queried with DEGs (*n* = 227). Of the 227 DEGs, 33 DEGs have been reported to be differentially methylated in peripheral blood of SCZ affected individuals (Kinoshita et al., 2014; Hannon et al., 2016; Montano et al., 2016). Specifically, Montano C et al. have reported differential methylation of *CCNE1, MCM3, AGO2, SCAP, TGFA* in SCZ participants which were in common to the 227 DEGs compiled in this review (Montano et al., 2016). Similarly, Hannon et al. reported *ZNF74, SLC45A1, ACOT7, GOT2, PRICKLE2, C11orf49, CARD11, NRG1, CD81, DISC1* and *AGO2* to be differentially methylated in SCZ (Hannon et al., 2016). Also, 22 differentially methylated genes reported by Kinoshita M et al. (viz. *RUNX3, ARF1, CD81, ZBTB4, SLC18A1, SALL1, RNPS1, KIF23, SIGIRR, CD6, GOT2, UBAP2L, PRKAB2, RAB11FIP3, SRPK1, NAF1, BRCA1, MMP9, SLC18A1, CHRNB2, ACOT7, LDB1*) were in common to the DEGs identified in this study (Kinoshita et al., 2014). AKT1 and BRCA1, the DEGs identified in this study have also been reported to be differentially methylated in brain prefrontal cortex (Wockner et al., 2014). Even with the limited number of epigenomics studies an overlap of differential methylation with expression was noteworthy. Interestingly, 11 of the 33 differentially methylated genes mentioned above (*ACOT7, AGO2, DISC1, LDB1, RUNX3, SIGIRR, SLC18A1, NRG1, CHRNB2, PRKAB2, and ZNF74)* were also associated with genetic alterations in SCZ. The above observation suggests that both genetic and epigenetic changes may result in differential expression of genes in SCZ.

### Comparative Gene Expression Analysis

**Figure 3:**
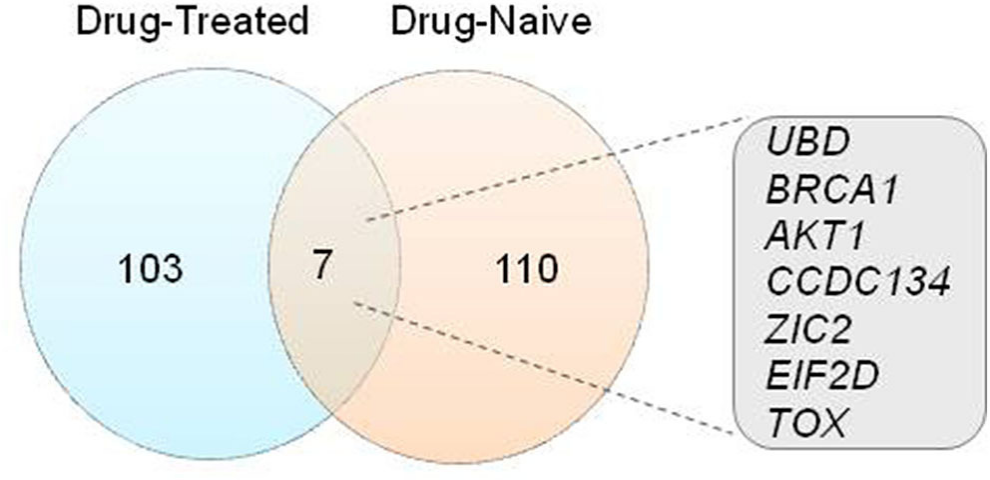
Comparative gene expression analysis. Visual representation of the relationship between 227 DEGs from microarray studies using Venn diagram analysis.

Out of the 227 DEGs identified from the 14 shortlisted microarray studies, 110 DEGs belong to the studies with drug-treated participants while 117 belonged to the studies with drug-naive participants (Supplementary Table 2). Three independent follow-up studies reported a profound effect of antipsychotic treatment on gene expression levels (Kumarasinghe et al., 2013; Xu et al., 2016; Gilabert-Juan et al., 2019). For an overview of the reported DEGs, a comparative analysis was performed between studies on drug-treated participants and drug-naive participants. *UBD, BRCA1, AKT1, CCDC134, ZIC2, EIF2D* and *TOX* were identified as common DEGs in both the groups (Figure 3 and Table 2). This analysis highlighted DEGs associated with SCZ which can be tested for their application as biomarkers.

**Table 2:** Gene information for the common DEGs between drug-treated and drug-naive participants from the microarray studies.

**Gene symbol**	**Gene name**	**Location**
*UBD*	Ubiquitin D	6p22.1
*BRCA1*	BRCA1 DNA repair associated	17q21.31
*AKT1*	AKT serine/threonine kinase 1	14q32.33
*CCDC134*	Coiled-coil domain containing 134	22q13.2
*ZIC2*	Zic family member 2	13q32.3
*EIF2D*	Eukaryotic translation initiation factor 2D	1q32.1
*TOX*	Thymocyte selection associated high mobility group box	8q12.1

### Gene Enrichment Analysis

We performed a functional enrichment analysis using g:Profiler for the DEGs identified in the shortlisted transcriptomic studies. The entire list of 227 DEGs with respect to controls resulted in significant enrichment of the biological processes such as 4-hydroxyproline and proline metabolism. The similar set of genes was processed for pathway enrichment analysis using DAVID functional annotation tool. Arginine and proline metabolism and ErbB signaling were the top two enriched pathways with *p* < 0.05 (Table 3). A significant enrichment of 2-Oxocarboxylic acid metabolism was also observed. A similar functional enrichment analysis was carried out for the 110 DEGs from the studies on drug-treated participants, and for the 117 DEGs from the studies on drug-naive participants separately. A significant enrichment of arginine and proline metabolism pathways was observed in drug-naive participants whereas the ErbB signaling pathway was enriched in the drug-treated participants.

**Table 3:** Top significantly enriched biological processes and molecular pathways for DEGs (*n* = 227) using g:Profiler and DAVID GO.

**Source**	**Term**	**Adj. *p* value**
Gene ontology	Proline catabolic process	0.011
(biological processes)	4-hydroxyproline catabolic process	0.011
	4-hydroxyproline metabolic process	0.011
KEGG pathways	Arginine and proline metabolism	0.004
	ErbB signaling pathway	0.006
	Non-small cell lung cancer	0.006
	Chronic myeloid leukemia	0.015
	2-Oxocarboxylic acid metabolism	0.021
	Prostate cancer	0.030
	Endometrial cancer	0.032
	Pathways in cancer	0.036

*KEGG, Kyoto Encyclopedia of Genes and Genomes*.

### Potential Biomarkers

It is essential to test candidate biomarkers for their diagnostic potential before suggesting their clinical application. Nine out of the previously identified 61 studies systematically evaluated the identified biomarkers for their efficiency (Table 4).

**Table 4:** Studies on identification and evaluation of potential biomarkers.

**Authors**	**Method**	**Method used for analysis**	**Biomarkers identified**	**Method for evaluation**	**Evaluation of results**	**Drug status of participant**
Tsuang et al. (2005)	Microarray and qRT-PCR	Logistic regression analysis and neural network analysis (data not shown in the article)	*APOBEC3B, ADSS2, ATM, CLC, CLCF1, CTBP1, DIDO1, CXCL1, S100A9*	Receiver operating characteristic Curve (ROC) analysis	Accuracy: 95–97%	Drug treated
Middleton et al. (2005)	Microarray and qRT-PCR	Class Predictor algorithm (GeneSpring): Euclidian nearest neighbor method and support vector machine (SVM)	(50 candidate genes)	Accuracy testing	Accuracy (Nearest neighbor): 95 % Accuracy (SVM): 100%	Not available
Takahashi et al. (2010)	Microarray	Supervised, artificial neural network (ANN)	(14 predictor genes)	Sensitivity and specificity testing	Sensitivity: 82.4%, Specificity: 93.8%	Drug naïve
Maschietto et al. (2012)	Microarray	Stepwise linear discriminant function	*HERPUD1, HOXA13, CTNNA1, SULT1A1, PIK3R3, MALAT1*	Leave-one-out cross validation	Sensitivity:89.3 %, Specificity: 90 %	Drug treated
Vachev et al. (2015)	qRT-PCR	Delta Ct values used for classification	*FEZ1*	ROC analysis for expression changes in targeted gene	Sensitivity:55.2 %, Specificity: 87.5 %, AUC: 0.728	Drug free (Participants were medication free for 1 month before sampling)
Okazaki et al. (2016)	qRT-PCR	Multivariate logistic regression analysis	*CDK4, MCM7* and *POLD4*.	ROC analysis	AUC:0.801	Drug treated
Gilabert-Juan et al. (2019)	Cohort 1 (cross-sectional): Microarray qRT-PCR	Binary logistic regression analysis	Cohort 1: *EIF2D* and *TOX*	Wald test	Cohort 1: Accuracy: 86.5%	Cohort1: Drug treated
					Cohort 2: Accuracy (0): 69.1%	Cohort 2: Drug naïve (at recruitment)
			Cohort 2: *EIF2D*		Accuracy (3 Months): 70.2%	
	Cohort 2 (follow-up): RNA-Seq				Accuracy (1 Year): 69.8%	
Trossbach et al. (2019)	qRT-PCR	Arbitrary threshold for combined gene expression levels of RGS1 and CCL4	*RGS1* and *CCL4*	Sensitivity and specificity testing based on expression levels of target genes	Sensitivity: 97 %, Specificity: 27 %	Drug treated
Zhu et al. (2021)	qRT-PCR	Machine learning (ML) methods (ANN, extreme gradient boosting, SVM, decision tree, and random forest	*GNAI1, FYN, PRKCA, YWHAZ, PRKCB*, and *LYN*	ROC analysis for each differentially expressed gene.	Sensitivity: 1.000, Specificity: 0.895 AUC: 0.993, and Youden index: 0.895	Not available
				AUC, sensitivity and specificity for performance testing of ML models		

Majority of the studies tabulated above (Table 4) use computational methods for identification of biomarkers. The qPCR-based methods that test one or two genes are associated with poor sensitivity or specificity. Irrespective of the drug status and other variations in cohorts, most of the models seem to diagnose SCZ with high efficiency.

### Effect of Antipsychotic Treatment

It is well known that antipsychotics induce significant gene expression changes in peripheral blood. Multiple studies have evaluated the effect of antipsychotics by post-treatment follow-up of the drug-naive or drug-free SCZ affected individuals. Studies suggest that the effect of antipsychotics can be observed within one week of the treatment (Zhang et al., 2008) and can be persistent for the duration of the medication (Okazaki et al., 2016). We identified 13 studies (4-Microarray, 9-PCR based) aimed at evaluating the effect of antipsychotics on gene expression, from the previously identified sixty one studies of SCZ. The DEGs and the reported change in their expression levels before and after antipsychotic treatment were tabulated (Table 5). We came across 27 genes which were reported to be differentially expressed upon antipsychotic treatment. Sixteen genes (*RPS25, DISC1, SLC2A3, UBD, NRG1, BRCA1, AKT1, MAL, RXRA, CCDC134, ZIC2, NLN, DAAM2, DGCR6, EIF2D and MMP9)* out of these 27 were part of the 227 genes identified earlier. The period of treatment for these studies varied from 1 week to 2 years. Importantly, most of the studies used drug-naive participants as the baseline samples. However, the types of antipsychotics and dosage varied across studies and participants. A few studies have evaluated the effect of specific antipsychotics such as Risperidone, Haloperidol and Olanzapine on gene expression levels of target genes (Shariati et al., 2009; Ota et al., 2015). A few of the follow-up studies reported no change in the expression status of *AKT1* (Kumarasinghe et al., 2013; Xu et al., 2016), *DISC1* (Kumarasinghe et al., 2013), *HP* (Trossbach et al., 2019) and *EIF2D* (Gilabert-Juan et al., 2019) on antipsychotic treatment. Thus, indicating their stronger association with the disorder. These genes can be further explored for their potential application as biomarkers and drug targets for development of effective treatment.

**Table 5:** Effect of antipsychotic treatment on gene expression pattern in peripheral blood of SCZ affected individuals.

**Gene name**	**Gene symbol**	**Pre treatment**		**Post treatment**		**Chromosomal location**
Dopamine receptor D3 (Vogel et al., 2004)	*DRD3*		↓		–	3q13.31
Very low density lipoprotein receptor (Suzuki et al., 2008)	*VLDLR*	↓			↑	9p24.2
LDL receptor related protein 8 (Suzuki et al., 2008)	*LRP8*		–	↓		1p32.3
Neuregulin 1 (Zhang et al., 2008)	*NRG1*	↓			↑	8p12
Transforming growth factor beta receptor 2 (Numata et al., 2008)	*TGFBR2*		↑	↓		3p24.1
Neurolysin (Kuzman et al., 2009)	*NLN*	↑			↓	5q12.3
Disheveled associated activator of morphogenesis 2 (Kuzman et al., 2009)	*DAAM2*		↑		–	6p21.2
Solute carrier family 2 member 3 (Kuzman et al., 2009)	*SLC2A3*	↑		–		12p13.31
AKT serine/threonine kinase 1 (Kumarasinghe et al., 2013; Xu et al., 2016)	*AKT1*		↑↑		– ↑	14q32.33
Retinoid X receptor alpha (Kumarasinghe et al., 2013)	*RXRA*	↑		–		9q34.2
Matrix metallopeptidase 9 (Kumarasinghe et al., 2013)	*MMP9*		↑		–	20q13.12
DISC1 scaffold protein (Kumarasinghe et al., 2013)	*DISC1*	↑		↑		1q42.2
Ribosomal protein S25 (Kumarasinghe et al., 2013)	*RPS25*		↓		–	11q23.3
DiGeorge syndrome critical region gene 6 (Kumarasinghe et al., 2013)	*DGCR6*	↓		–		22q11.21
mal, T cell differentiation protein (Kumarasinghe et al., 2013)	*MAL*		↓		–	2q11.1
Myelin basic protein (Ota et al., 2015)	*MBP*	↑		–		18q23
nudE neurodevelopment protein 1 like 1 (Ota et al., 2015)	*NDEL1*		↑		–	17p13.1
BRCA1 DNA repair associated (Xu et al., 2016)	*BRCA1*	↑		–		17q21.31
Coiled-coil domain containing 134 (Xu et al., 2016)	*CCDC134*		–		↓	22q13.2
Ubiquitin D (Xu et al., 2016)	*UBD*	–		↓		6p22.1
Zic family member 2 (Xu et al., 2016)	*ZIC2*		–		↓	13q32.3
Cyclin dependent kinase 4 (Okazaki et al., 2016)	*CDK4*	↓		↑		12q14.1
Minichromosome maintenance complex component 7 (Okazaki et al., 2016)	*MCM7*		↓		–	7q22.1
DNA polymerase delta 4, accessory subunit (Okazaki et al., 2016)	*POLD4*	↓		↑		11q13.2
Haptoglobin (Yee et al., 2017)	*HP*		↑		↑	16q22.2
Eukaryotic translation initiation factor 2D (Gilabert-Juan et al., 2019)	*EIF2D*	↓		↓		1q32.1
Signal transducer and activator of transcription 3 (Subbanna et al., 2020)	*STAT3*		↑		↓	17q21.2

*The “↑” indicates up-regulation, “↓” indicates down-regulation and “–” indicates no change in the expression levels. The gene expression changes recorded in the post treatment column are with respect to the baseline expression levels and/or with respect to controls in the given study. The gene names and gene symbols mentioned in the table are HGNC approved*.

## Discussion

A significant number of gene expression studies have reported DEGs in association with SCZ. However, we did not come across any systematic review for gene expression studies aiming for biomarker discovery using peripheral blood. Here we attempt to summarize 61 gene expression studies on peripheral blood with special interest in microarray based expression profiling. The studies were based on diverse ethnic groups including Caucasian, Han Chinese and Japanese populations. Dedicated studies on Southeast Asian and African, and other populations were fewer (Glatt et al., 2009; Bousman et al., 2010b; Kumarasinghe et al., 2013; Yee et al., 2017). This bias needs to be addressed since more studies from diverse populations will be helpful in establishment of robust biomarkers for SCZ. As mentioned earlier, majority of the identified studies were cross-sectional in nature. Cross-sectional studies can provide important insight into the stage of the disease at a given point of time. However, in case of a multiform disorder like SCZ which is known to progress in more than one defined direction, it is important to have a prospective cohort study for biomarker discovery. Prospective studies with clinically high-risk participants can also help in identifying underlying biological processes associated with SCZ endophenotypes.

A well-designed cohort study can provide significant leads to the discovery of biomarkers for SCZ. Accurate sample size, uniform clinical diagnosis and extensive clinically relevant information (e.g. smoking, drug abuse, Body Mass Index, years lived with disorder, age of onset of the disorder, family history, prescribed medication, nutritional status, etc.) are of paramount importance for establishing a cohort for the discovery of biomarkers. A very few studies from our list provided such elaborate information (Bousman et al., 2010a; Glatt et al., 2011; Gardiner et al., 2013). Further to address the diversity in the clinical symptoms of SCZ, symptom-based correlation of biomarkers is a necessity. Unlike any other complex disorders, SCZ phenotypes may exhibit a different set of symptoms with different intensities at the onset of the disorder. Hence, clinical interviews and symptom rating scales (e.g., PANSS and BPRS) gain significant importance in the cohort establishment. Though, the majority of the studies shortlisted by us used DSM for diagnosis, only a few used structured clinical interviews for the same. A routine use of clinical interviews and rating scales can be helpful in identification of more accurate biomarkers for SCZ and its severity.

In the past couple of decades, the use of microarray technology has picked up a pace and has provided compelling evidence for its application in biomarker discovery. However, the use of cutting-edge technologies like Next Generation Sequencing (NGS) remains scanty for transcriptomic studies for SCZ compared to their use in genetic studies. Unlike microarray technology, NGS is less affected by technical problems such as background noise and data normalization. We came across only two studies based on RNA sequencing (RNA-Seq) to identify gene expression changes in peripheral blood (Gilabert-Juan et al., 2019; Zhang et al., 2020). Gilabert-Juan et al., reported differential expression of EIF2D at onset of the disorder, and at 3 months and 1 year post treatment follow-up compared to controls (Gilabert-Juan et al., 2019). A study by Zhang et al., identified co-expressed genes associated with abnormal psychomotor behavior in SCZ (Zhang et al., 2020). Also, of the 506 DEGs reported by Zhang et al., *SRPK1, DEFA4, ACO1* were common to the 110 DEGs from microarray studies with drug treated participants and *UBAP2L, TKTL1, KIF23* were common to the 117 DEGs from microarray studies with drug naive participants identified in this literature survey. A large number of whole-genome transcriptomic studies using NGS can accelerate the biomarker discovery for psychiatric disorders. NGS based studies will not only provide better insight into functional genomics of the SCZ but will also generate a substantial number of leads which can later be validated for their application as biomarkers.

As described earlier, we screened 14 microarray-based gene expression studies in SCZ for analysis. We categorized these studies into “drug treated” and “drug naïve” groups and compiled the gene expression changes. We found 7 common genes (viz. *AKT1, EIF2D, CCDC134, ZIC2, TOX, UBD*, and *BRCA1*) between these two groups suggesting their potential role as biomarkers irrespective of the drug status (Figure 3). In total, four studies independently reported differential expression of AKT1 (Kumarasinghe et al., 2013; Liu et al., 2016; Xu et al., 2016; Mostaid et al., 2017). A diagnostic accuracy of 86.5% for EIF2D and TOX was reported using Weighted Gene Co-Expression Network Analysis (WGCNA) and predictive mathematical model (Gilabert-Juan et al., 2019). These studies suggest that the common DEGs in drug-treated and drug-naïve groups hold a promise for diagnosis irrespective of the medication status of the participants. Also, additional information about the drug status may help in improving the accuracy of the diagnosis. Various computational methods have also been used for development of diagnostic models. Generally, classification methods based on fewer genes suffer from relatively poor sensitivity or specificity of diagnosis (Vachev et al., 2015; Trossbach et al., 2019). The artificial neural network based methods seem to improve the accuracy of diagnosis (Takahashi et al., 2010; Zhu et al., 2021); however, they often lack the “explanability” required for clinical applications. Development of explainable learning based models can be crucial in this aspect.

A few studies have also identified transcriptional changes in the first degree siblings and discordant twins in comparison to their affected counterparts (Middleton et al., 2005; Glatt et al., 2011; Zheutlin et al., 2016). The same studies also pointed out gene expression differences between unaffected siblings and controls. Similarly, a PCR based study reported over expression of TH, IL-1β and TNF-α in both SCZ affected participants and their siblings when compared to controls (Liu et al., 2010). This suggests the use of these DEGs as biomarkers for inherited vulnerability. On similar lines, Ota et al., identified transcriptional changes which could differentiate clinical high-risk participants, first episodic individuals and participants with chronic SCZ from each other (Ota et al., 2019). Genetic and epigenetic studies component of SCZ have identified a large number of quantitative trait loci and differentially methylated genes in SCZ. A significant number of DEGs identified in this study were reported earlier to be differentially methylated in peripheral tissue such as blood. As mentioned earlier, we found an overlap in the differentially expressed genes and the genetic loci associated with SCZ. Altogether, the findings suggest that the genetic and epigenetic changes associated with SCZ can influence the gene expression in peripheral tissue such as blood. These gene expression changes can offer more specificity as biomarkers for the diagnosis of SCZ at an early stage of the disease.

We further performed a gene enrichment analysis using 227 DEGs compiled from microarray studies. Enrichment of arginine, proline metabolism pathway in our analysis is in agreement with the findings of the recent metabolomics study which reported changes in amino acid signatures in SCZ affected individuals (He et al., 2012; Parksepp et al., 2020). In addition to this a study by Chen et al. reported arginine and proline metabolism as one of the differential metabolic pathways in violent schizophrenia affected participants (Chen et al., 2020). Interestingly, 4-hydroxyproline was identified as one of the potential metabolic markers by this study (Chen et al., 2020). These findings are in accordance to our observations where 4-hydroxyproline was one of the enriched metabolic processes in the gene enrichment analysis (Table 3). Similarly, enrichment of ErbB signaling pathway and differential expression of NRG1 in our compiled data suggests the dysregulated NRG-ErbB pathway in SCZ participants. This alteration in NRG-ErbB pathway in SCZ has been reported earlier in independent studies as well (Wu et al., 2016; Mostaid et al., 2017).

Besides the enriched terms and pathways identified by our analysis, metabolomic studies have also identified impaired glucose and lipid metabolism in association with SCZ (Orešič et al., 2011; Liu et al., 2015). Two independent pathway analyses, irrespective of medication status, have highlighted a significant enrichment of pathways regulating the immune response (Gardiner et al., 2013; Wu et al., 2016). Recently, the KEGG-based pathway analysis by Xu et al. identified olfactory transduction and protein digestion and absorption as enriched pathways in drug-naive participants (Xu et al., 2016). Similarly, Kumarasinghe et al. reported enrichment of pathways involving AKT1 signaling in drug-naive participants (Kumarasinghe et al., 2013). Glutamate metabolism, chondroitin sulfate biosynthesis (Bousman et al., 2010a), and neural signaling pathways (Wu et al., 2016) on the other hand have been found to be enriched in drug-treated participants with SCZ. The glucose and lipid metabolism are suspected to be dysregulated due to antipsychotic medication and unhealthy lifestyle (Henderson et al., 2005; Ventriglio et al., 2015). However, the dysregulated amino acid metabolism, immune pathways, AKT and ErbB signaling are suspected to be involved in pathogenesis of the SCZ and thus can be further explored as potential targets for drug discovery and biomarker research (Buonanno, 2010; Zheng et al., 2012; Saleem et al., 2017; Van Kesteren et al., 2017).

In an attempt to identify the effect of the DEGs associated with antipsychotic treatment we screened 13 follow-up studies (9-PCR, 4-microarray) from the previously identified 61 studies. The tabulation of gene expression status of the DEGs before and after treatment revealed that the antipsychotic treatment does influence peripheral blood gene expression in SCZ, thus, suggesting the importance of drug-naive participants in diagnostic biomarker discovery for SCZ. Of the 27 genes identified from the follow-up studies only *AKT1, DISC1* (Kumarasinghe et al., 2013; Xu et al., 2016), *HP* (Trossbach et al., 2019) and *EIF2D* (Gilabert-Juan et al., 2019) had no effect of antipsychotic treatment on their expression status (Table 5). Therefore, these genes can be further explored for their potential application as diagnostic biomarkers. Also, the genes whose expression status is influenced by antipsychotic treatment can serve as potential candidates for predictive biomarker discovery (Table 5). However, these findings need further validation based on multi-time point follow up studies.

The recent gene expression studies in SCZ have provided sufficient evidence for their potential use as biomarkers to support the current diagnosis. Along with ease of detection, gene expression can offer valuable insight into the complexity of the disorder. Recent developments in sequencing technology have a lot to offer for biomarker discovery in SCZ. Use of microarray for biomarker discovery has resulted in generation of a significant amount transcriptomic data. These datasets can prove useful for the meta-analysis project to identify significant potential hits for the discovery of biomarkers. However, a very few researchers make their raw data publicly available. Of the microarray studies which are reviewed in this article, only one research group (Bousman et al., 2010b) has deposited their data on Gene Expression Omnibus (GEO) (Barrett et al., 2013). Discovery of gene expression biomarkers for SCZ needs integration of clinical psychiatry tools, statistical analysis, transcriptomic datasets, bioinformatics pipeline, and artificial intelligence-based predictive modeling. A collaboration of the experts from the respective field is the key to the discovery of biomarkers for Schizophrenia.

## Conclusion

Here we reviewed the potential of DEGs to be used as biomarkers at various stages of schizophrenia. The unprecedented insight offered by the DEGs into the complexity of schizophrenia and the ease of their application into clinics outweighs other proposed molecular biomarkers. Our preliminary functional analysis of the previously reported DEGs sheds light on arginine, proline and hydroxyproline metabolism in association with SCZ. According to our literature survey, AKT1 remains the most frequently reported differentially expressed gene associated with SCZ even with many diverse study designs and detection techniques. The current literature provides sufficient evidence for the existence of specific gene expression patterns for SCZ. The use of NGS (RNA-Seq) and machine learning approaches is yet to be exploited for the detection of robust biomarkers. Further, the efforts made toward establishing prospective cohorts of the younger population with the multi-omics approach will contribute substantially toward the discovery of gene expression biomarkers for SCZ.

## Data Availability Statement

The original contributions presented in the study are included in the article/Supplementary Material, further inquiries can be directed to the corresponding authors.

## Author Contributions

VW and SK constructed the theme and structure of the research article. VW, SK, and VP wrote the manuscript. VP and SA provided inputs on the clinical aspects of the review. VW extracted and compiled the data. TP and PV verified the data. All authors have read and approved the manuscript.

## Funding

The study was funded by an intramural research grant (MjRP/19-20/1516) from Symbiosis Center for Research & Innovation (SCRI), SIU, Pune, India. VW and PV received the research fellowships from UGC, New Delhi, India and MjRP, Symbiosis Center for Research & Innovation (SCRI), SIU, Pune, India respectively. SK is also a beneficiary of a DST SERB SRG grant (SRG/2020/001414).

## Conflict of Interest

The authors declare that the research was conducted in the absence of any commercial or financial relationships that could be construed as a potential conflict of interest.

## Publisher's Note

All claims expressed in this article are solely those of the authors and do not necessarily represent those of their affiliated organizations, or those of the publisher, the editors and the reviewers. Any product that may be evaluated in this article, or claim that may be made by its manufacturer, is not guaranteed or endorsed by the publisher.

## Footnotes

^1^The term percentage of heritability here does not represent the causal rate of the disorder but the variation observed in traits within the given population (Tenesa and Haley, 2013; Moore and Shenk, 2017).
